# Characterization of Bacterial and Fungal Assemblages From Historically Contaminated Metalliferous Soils Using Metagenomics Coupled With Diffusion Chambers and Microbial Traps

**DOI:** 10.3389/fmicb.2020.01024

**Published:** 2020-06-10

**Authors:** Ashish Pathak, Rajneesh Jaswal, Xiaoyu Xu, John R. White, Bobby Edwards, Jaden Hunt, Scott Brooks, Rajesh Singh Rathore, Meenakshi Agarwal, Ashvini Chauhan

**Affiliations:** ^1^School of the Environment, Florida A&M University, Tallahassee, FL, United States; ^2^Savannah River Ecology Laboratory, University of Georgia, Aiken, SC, United States; ^3^Department of Oceanography and Coastal Sciences, Louisiana State University, Baton Rouge, LA, United States; ^4^Environmental Sciences Division, Oak Ridge National Laboratory, Oak Ridge, TN, United States

**Keywords:** mercury resistance, metagenomics, diffusion chamber, microbial trap, culturomics

## Abstract

The majority of environmental microbiomes are not amenable to cultivation under standard laboratory growth conditions and hence remain uncharacterized. For environmental applications, such as bioremediation, it is necessary to isolate microbes performing the desired function, which may not necessarily be the fast growing or the copiotroph microbiota. Toward this end, cultivation and isolation of microbial strains using diffusion chambers (DC) and/or microbial traps (MT) have both been recently demonstrated to be effective strategies because microbial enrichment is facilitated by soil nutrients and not by synthetically defined media, thus simulating their native habitat. In this study, DC/MT chambers were established using soils collected from two US Department of Energy (DOE) sites with long-term history of heavy metal contamination, including mercury (Hg). To characterize the contamination levels and nutrient status, soils were first analyzed for total mercury (THg), methylmercury (MeHg), total carbon (TC), total nitrogen (TN), and total phosphorus (TP). Multivariate statistical analysis on these measurements facilitated binning of soils under high, medium and low levels of contamination. Bacterial and fungal microbiomes that developed within the DC and MT chambers were evaluated using comparative metagenomics, revealing *Chthoniobacter*, *Burkholderia* and *Bradyrhizobium* spp., as the predominant bacteria while *Penicillium*, *Thielavia*, and *Trichoderma* predominated among fungi. Many of these core microbiomes were also retrieved as axenic isolates. Furthermore, canonical correspondence analysis (CCA) of biogeochemical measurements, metal concentrations and bacterial communities revealed a positive correlation of *Chthoniobacter*/*Bradyrhizobium* spp., to THg whereas *Burkholderia* spp., correlated with MeHg. *Penicillium* spp., correlated with THg whereas *Trichoderma* spp., and *Aspergillus* spp., correlated with MeHg, from the MT approach. This is the first metagenomics-based assessment, isolation and characterization of soil-borne bacterial and fungal communities colonizing the diffusion chambers (DC) and microbial traps (MT) established with long-term metal contaminated soils. Overall, this study provides proof-of-concept for the successful application of DC/MT based assessment of mercury resistant (HgR) microbiomes in legacy metal-contaminated soils, having complex contamination issues. Overall, this study brings out the significance of microbial communities and their relevance in context to heavy metal cycling for better stewardship and restoration of such historically contaminated systems.

## Introduction

Despite advancements in the area of culturomics, which is the coupled application of omics-based tools, such as metagenomics, to improve cultivation efficiencies of environmental microbiota, the vast majority of soil microorganisms remain uncultivable (Keller and Zengler, 2004; Vartoukian et al., 2010; Locey and Lennon, 2016; Hofer, 2018; Hahn et al., 2019; Bodor et al., 2020), and hence, unavailable for downstream environmentally relevant applications, such as bioremediation. Some of the major factors that preclude successful isolation of environmentally and functionally relevant microbiomes have been reviewed in a recent article (Bodor et al., 2020) and include soil dilution- eliminating the microbes that occur in extremely low numbers; nutrient-rich media components- facilitating rapidly growing microbes to outcompete the slow growing microbes that may provide critical ecosystem services; and the absence of cofactors produced by microbial communities in their native habitat (Nichols et al., 2008), that are lacking in standard laboratory cultivation techniques. There are several strategies that can be employed to improve microbial growth and isolation, especially from environmental samples, such as varying media composition (Joseph et al., 2003); high throughput extinction culturing (Rappé and Giovannoni, 2003); high throughput single-cell encapsulation (Zengler et al., 2002), diffusion chambers (Bollmann et al., 2010); using different gelling agents, antioxidants, or signaling molecules (Bruns et al., 2002; Tamaki et al., 2009); increasing incubation times (Davis et al., 2005); preparing the growth media with simple alterations (Kato et al., 2018); and even extracting soil nutrients to make media (Nguyen et al., 2018). Furthermore, metagenomic analysis coupled with aforementioned cultivation techniques, can serve as a sensitive and precise tool to assess the environmental microbiomes (bacterial) and mycobiomes (fungal); which can then be isolated using appropriate strategies for downstream applications.

We have successfully optimized the diffusion chamber (DC) and microbial trap (MT) techniques, as reported elsewhere (Berdy et al., 2017), and have advanced our understanding on uranium (U)-resistant bacterial and fungal assemblages in soils with long-term history of metal contamination (Jaswal et al., 2019a, b). To establish diffusion chambers, soils are serially diluted, mixed with sterile agar, and inoculated in between two semipermeable membranes of variable pore sizes, depending on the microorganisms being targeted for isolation, i.e., bacteria, actinomycetes or fungi. The chambers are then placed on the same soils (moistened if necessary), from which dilutions were carried out, thus permitting continuous exchange of chemicals and nutrients from the soils into the overlaying chamber via diffusion while restricting the movement of microbial cells from the chamber to the external environment. Biomass entrapped within the chambers is thus enriched by the soil nutrients and after appropriate incubation times, chambers are opened, biomass is collected (referred to as generation 1), and if needed, transferred to new chambers (referred to as generation 2), to enhance recovery of environmentally relevant isolates (Bollmann et al., 2007, 2010). The microbial trap (MT) method, on the other hand, has been specifically developed to enhance growth and isolation of soil-borne actinomycetes and/or fungi (Gavrish et al., 2008) (Supplementary Figure S1), in that the bottom membrane, which lies in close proximity to the soil, consists of 0.2 μm pore size; thus, permitting the entry of fungal hyphae but not bacteria into the trap.

Our previous studies on the application of DC and MT approaches to heavy-metal contaminated soils have yielded interesting findings (Jaswal et al., 2019a, b), which are summarized as follows: (1) DC/MT growth chambers resulted in the enrichment of environmentally relevant microbiota fed only by soil nutrients that diffused into the chambers, thus circumventing the use of artificial growth media which is an inherently biased cultivation approach; (2) the top genera, also called as the core diversity, which are dominant at threshold levels of 20% (sample prevalence) and 0.2% relative abundance, in the previous DC and MT experiments were identified by metagenomics to taxonomically affiliate with bacterial genera of *Burkholderia*, *Bradyrhizobium*, and *Rhodanobacter*; among fungi, *Penicillium* and *Trichoderma*, predominated; (3) the abundance and diversity of the desired core microbiomes significantly increased as a function of enrichments over three consecutive transfers (aka generations) of DC and MT; (4) metagenomic analysis of the DC/MT generations revealed that enrichment and stable populations of the core bacterial and fungal microbiomes developed within the first 20 days of incubation; (5) the core microbiomes were subsequently retrieved as axenic isolates and possessed high resistance abilities against uranium (U) and by inference, these isolates were found to be functionally and environmentally relevant in context to U bioremediation; (6) using the MT approach, we successfully isolated a potentially novel *Penicillium* species, as evidenced by whole genome sequence analysis and comparative genomics, thus enhancing our overall understanding on the uranium cycling microbiota within the tested uraniferous soils and validating the use of DC/MT as a powerful culturomics technique (Jaswal et al., 2019a, b).

In this study, we coupled the application of DC and MT with amplicon-based metagenomics on soils obtained from two different locations characterized with long-term history of contamination with heavy metals such as mercury (Hg). Study sites included the United States Department of Energy managed Savannah River Site (SRS), located near Aiken, South Carolina and Oak Ridge Reservation (ORR), located in Oak Ridge, Tennessee. Mercury contamination in the ORR and SRS sites are still pervasive with soil concentrations ranging from 9.80 ng/g to 1688 ng/g, as shown in this and other studies (Brooks and Southworth, 2011; Xu X. et al., 2019). Note that Hg is a naturally occurring environmental element but is toxic and exists in several forms under environmental conditions including inorganic ionic and elemental forms (Hg^2+^, and Hg(0), respectively) as well as microbially produced organic forms (e.g., monomethylmercury, MeHg). Poisoning by inorganic Hg^2+^ is reversible with chelation treatment (Chen et al., 2006), but on the other hand, MeHg, which is both neurotoxic and carcinogenic, can bioaccumulate and bio-magnify in the food chain, and can cause irreversible poisoning (Sweet and Zelikoff, 2001; Desrosiers et al., 2006). Anthropogenic activities have severely perturbed the natural Hg environmental cycle mobilizing Hg from long-term geologic storage into the active atmospheric, shallow surface terrestrial and aquatic environments (Selin, 2009). Once in aquatic environments, biotic reactions can convert inorganic mercury (Hg^2+^) to methylmercury (Pak and Bartha, 1998), exerting negative impacts to both, ecological processes and public health. Some bacteria present in Hg-rich environments express Hg resistance (HgR) by transforming mercury compounds (inorganic and organic) to Hg vapor (Nakamura et al., 1988, 1990), and are broadly classified as Hg resistant (HgR). Highly resistant bacteria may be able to volatilize both inorganic Hg(II) and organic Hg compounds, whereas some other bacteria can volatilize only Hg(II) (Clark et al., 1977; Weiss et al., 1977). Hg cycling bacterial and fungal communities are now much better understood with the application of metagenomic techniques (von Canstein et al., 2002; Gionfriddo et al., 2016). Well known HgR bacteria mainly belong to the phyla Proteobacteria, Firmicutes and Actinobacteria (Nazaret et al., 2003; Duran et al., 2008; Vishnivetskaya et al., 2010; Xu J. et al., 2019; Pathak et al., 2020); along with Verrucomicrobia and Epsilonproteobacteria, which were also observed in MeHg contaminated sediment samples from the U.S. Department of Energy’s Field Research Center (FRC) in Oak Ridge, TN (Vishnivetskaya et al., 2010). The HgR bacteria reported thus far include nine genera: *Aeromonas*, *Bacillus*, *Burkholderia*, *Escherichia*, *Pseudomonas*, *Staphylococcus*, *Stenotrophomonas*, *Streptococcus*, and *Tolumonas*, respectively. More recently, we used comparative genomics to characterize another HgR strain that belongs to *Arthrobacter* genera, which contained the mercuric ion reductase (merA), the organomercurial lyase (merB), and the mercuric resistance operon regulatory protein, isolated from the H-02 wetland soils also included in this study (Pathak et al., 2020). Moreover, several HgR fungi, mainly belonging to the Ascomycota phylum, such as *Candida* sp. and *Pichia* sp. were isolated from wastes and sewage water in Lahore, Pakistan (Amin and Latif, 2011). Fungi belonging to genera *Aspergillus*, *Cladosporium*, *Trichoderma*, and *Alternaria* isolated from a Hg mining plant in Rudňany in central Slovakia, were resistant to 32 mg/L Hg (Urík et al., 2014). Note that it is not only imperative to identify the environmentally relevant microbiota, which in this study pertain to HgR, but these microbiotas also need to be isolated for subsequent environmental applications.

Note that it is not merely the microbial communities that exert methylation of inorganic Hg to methylmercury (MeHg), but this is also a function of several other environmental factors that includes bioavailability of inorganic Hg(II) to methylating microbiota as well as the competing Hg demethylation process wherein the presence/activity of demethylating bacteria can convert MeHg into less toxic forms by cleaving the carbon-mercury bond (Hg-CH_3_) (Hsu-Kim et al., 2013). Therefore, a suite of coexisting microbial, biogeochemical and environmental conditions dictates the net rate of MeHg production, with the main environmental controllers being organic matter (Deonarine and Hsu-Kim, 2009), sulfate concentrations (Benoit et al., 1999; Xu X. et al., 2019), pH and temperature (Hsu-Kim et al., 2013). In this context, there have been numerous studies to examine the role of dissolved organic matter (DOM) on the bioavailability and fate of Hg at the ORR in Tennessee (Miller et al., 2009; Dong et al., 2010); here DOM concentrations typically vary between 2.5 and 3.5 mg/L. Even at this relatively low DOM concentration, Hg complexation to sulfate or thiol-like functional groups of DOM exerted a strong control on Hg speciation (Dong et al., 2010). Therefore, the significance of Hg interaction with DOM at a historically contaminated ORR site has been well established; not as much information is known on HgR strains and processes in the SRS impacted habitats.

Interesting insights into the bacterial and fungal communities inhabiting Hg-rich environments have been made. One application of this knowledge is to manipulate the community composition or enrich for those strains that can mediate a desired function such as environmental restoration. To that end, identifying the environmentally relevant microbiota, which can be considered to be the HgR and mercury demethylators for Hg bioremediation, and their subsequent isolation for additional studies and applications is essential (Dash and Das, 2012). Following their isolation, further studies using these model groups of microbiotas can then be performed to obtain a deeper understanding on the microbial fate of environmental contaminants, such as mercury (Hg) in historically Hg-rich soils. Because we have successfully demonstrated the application of diffusion chambers (DC)/microbial traps (MT) to characterize the heavy metal resistant microbiota in uranium contaminated SRS soils, the goal for this study was to extend the DC/MT culturomics approach to study mercury resistant (HgR) bacteria and fungi from soils with variable levels of Hg contamination. To do so, we collected soils with high, medium and low levels of Hg contamination and established DC/MT chambers followed by assessment of shifts in the microbial assemblages as a function of *in situ* enrichment within the DC/MT growth chambers. Overall, the overarching objective was to couple the application of metagenomics to assess the bacterial and fungal assemblages colonizing within the DC/MT chambers followed by isolation of the functionally and environmentally relevant HgR strains. The next objective was to use the isolated strains as model organisms and evaluate their abilities to resist Hg. To the best of our knowledge, this is the first report on the application of diffusion chambers/microbial traps for the isolation and characterization of Hg resistant microbiota from legacy contaminated soils.

## Materials and Methods

### Site Description and Sample Collection

**FIGURE 1:**
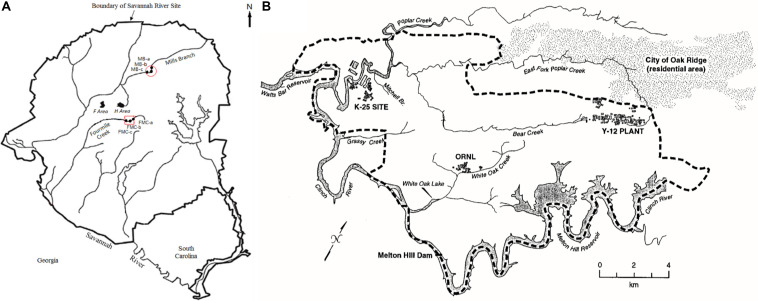
Map of soil sampling locations included in this study. **(A)** Savannah River Site (SRS), Aiken, South Carolina, and; **(B)** Oak Ridge Reservation (ORR), Tennessee. Maps shown here have been previously reported by Xu X. et al. (2019) (SRS) and Campbell et al. (1998) (ORR), respectively.

Two legacy metal-contaminated sites were chosen for this study- the Savannah River Site (SRS) (Figure 1A, Xu X. et al., 2019), located near Aiken, South Carolina and East Fork Poplar Creek (EFPC) on the ORR (Figure 1B, Campbell et al., 1998), located in Oak Ridge, Tennessee. Both these sites have locations characteristic of long-term history with heavy metal and organic contaminants (Brooks and Southworth, 2011). Additionally, a reference site from the SRS (R1), was also chosen that has no direct contamination history, other than atmospheric deposition of Hg. GPS coordinates of the sampled locations are listed in Table 1.

**TABLE 1:** Biogeochemical, tHG and MeHg concentrations measured from the collected soil samples from Savannah River Site (SRS) and Oak Ridge Reservation (ORR), respectively.

Site	GPS Location of Sampled Site	Moisture Content (wt. %)	Total Carbon (g/kg)	Total Nitrogen (g/kg)	Total Phosphorus (mg/kg)	Loss on Ignition (wt. %)	Extractable NH^+^_4_ (mg/kg)	Extractable NO^–^_3_ (mg/kg)	Extractable PO_4_^3–^ (mg/kg)	Total Mercury (ng/g)	Methyl Mercury (ng/g)
H-02	33.1727/−81.3852	54.8	2.868	1.89	308	6.5	32.13	3.230	0.096	9.80	0.27
S1	33.167806/−81.746851	69.0	5.997	4.469	1371	20.0	114.19	0.623	0.335	788.28	0.98
S3	33.167806/−81.746851	50.5	4.156	2.245	242	8.0	4.99	0.250	b.d.	26.19	0.82
R1	33.163340/−81.745884	32.8	1.219	0.628	68.5	2.4	29.67	b.d.	b.d.	13.64	0.27
B	35.5758/−842128	31.8	0.951	0.852	495	4.3	61.81	0.550	0.027	1688.31	1.21

*Some values were below detection (bd).*

SRS consists of an approximately 800-km^2^ area, which housed a former nuclear weapons production facility located along the Savannah River near Aiken, SC, United States (Sowder et al., 2003). SRS is of significant interest because of widespread contamination by residual heavy metals and radionuclides from the previous DOE weapons production activities. SRS environmental monitoring has revealed that the groundwater and riparian sediments contain uranium (U), nickel (Ni) and other metals such as mercury (Hg), specifically in the M-Area (Sowder et al., 2003; Figure 1A). Tims Branch, which drains the upland region around the M area into Upper Three Runs and eventually the Savannah River, is a second-order stream that receives contamination from Steeds Pond, a former farm pond that served as a radiological setting basin. The U.S. Department of Energy’s 14,013-ha (34,600-acre) ORR in eastern Tennessee is the other site included in this study, which houses three main facilities (the Oak Ridge Y-12 Plant, the K-25 Site, and Oak Ridge National Laboratory, Figure 1B); some of these sites were placed on the National Priorities List as a Comprehensive Environmental Response, Compensation, and Liability Act (CERCLA) site requiring investigation in 1989 (Ferrari et al., 2005).

From the SRS, sub-surface soil samples were collected from an abandoned pond (Steeds Pond), that served as a natural settling basin along the Tims Branch stream corridor. Four different locations at this site were selected on the basis of previous studies that reported variable levels of Hg contamination, namely S1 (medium Hg levels), H-02 (low Hg levels), R1 (low Hg levels), and S3 (low Hg levels). Sites S1 and S3 are from within the Savannah River Swamp System (SRSS), which is a flood plain and a hotspot of Hg contamination (Xu X. et al., 2019), located at the confluence of Fourmile Creek and Savannah River. Note that one of the biggest sources of Hg contamination was a chloralkali facility near Augusta, GA, United States, that released approximately 18,000 lbs. of Hg into the Savannah River (Xu X. et al., 2019). Concurrently, the seepage basins at F and H Areas on the Savannah River Site received liquid effluents that contained Hg until the 1980s, which seeped through the ground and outcropped in the wetlands. Site H-02 is a constructed wetland system that treats storm water runoff from the Tritium Processing Facility at SRS (Xu and Mills, 2018). Samples from site R were collected from the Mills branch, which received Hg from atmospheric deposition and historical agricultural run-off (Newman, 1986).

The EFPC, in Oak Ridge, TN, United States, characterized by high levels of Hg contamination (Brooks and Southworth, 2011). Specifically, stream sediment samples were obtained from the EFPC, labeled as sample ‘B.’ At the EFPC site, contamination is associated from past discharges, spills, and accidents from industrial processes at the creek headwaters. Estimated total mercury released to EFPC ranges from 93 to 163 metric tons (Campbell et al., 1998). Although the primary mercury losses stopped in 1963, mercury continues to be released into EFPC from secondary sources (contaminated buildings, equipment, and soils) at approximately 5.3 g/d.

Soil samples collected from Tims Branch, Mills Branch and EFPC were stored on ice and shipped to the FAMU laboratory, where they were processed immediately upon receipt, to establish diffusion chambers (DC) and microbial traps (MT). Chemicals used in this study are analytical grade and purchased from VWR (Atlanta, GA, United States), unless otherwise stated.

### Mercury and Biogeochemical Measurements

For biogeochemical analyses, soil samples were collected in duplicate from the above stated reference, low, medium and high Hg-contaminated locations and were independently analyzed. Soil moisture content (MC) was obtained using the standard gravimetric method (Vaccare et al., 2019). Total carbon (TC) and total nitrogen (TN) were determined on dried, ground subsamples using a Carlo-Erba NA-1500 CNS analyzer (Haak-Buchler Instruments, Saddlebrook, NJ, United States) (Sapkota and White, 2019). For total phosphorus (TP), 0.5 g of dried, ground sample was combusted at 550°C for 4 h in a muffle furnace, followed by dissolution of the ash in 6 M HCl on a hot plate (Sapkota and White, 2019). Total P was analyzed in the digested solution using an automated ascorbic acid method using a Seal AQII discrete analyzer (Mequon, WI, United States), according to the U.S. EPA method 365.4 (Pfaff, 1993). Extractable nutrients were determined using a 2 M KCL extraction of a moist subsample, passed through a 0.45 μm membrane filter and preserved with acid to a pH of below 2 (Steinmuller et al., 2020). The extracts were analyzed on a Seal AQII discrete analyzer, using standard U.S. EPA colorimetric methods (Pfaff, 1993).

Total Hg (THg) concentrations in sediment samples were analyzed by atomic absorption spectrophotometry with a Direct Mercury Analyzer-80 (Milestone; Shelton, Connecticut, USA) on a dry-weight basis and expressed as ng/g, as shown before (Xu X. et al., 2019). Standard curves were obtained using a standard reference material within each analytical run; precision and accuracy of the analytical system were quantified by running blank samples, replicated samples, as well as certified standard reference materials. Methyl-Hg (MeHg) concentrations in sediment samples were analyzed by MERX Automated Methylmercury System (Brooks Rand, Seattle, WA, United States). Prior to the methyl-Hg analysis, sediments were prepared by acid leaching, solvent extraction, and water back extraction processes (Liang et al., 2004). This was followed by buffering appropriate aliquots of prepared samples with sodium acetate and ethylated by sodium tetraethylborate (Liang et al., 1994), followed by quantification of methyl-Hg using gas chromatographic separation and pyrolysis analysis using cold vapor atomic fluorescence. Calibration curves were obtained using a liquid methyl-Hg standard CH_3_HgCl (Brooks Rand, Seattle, WA, United States) that was included with each analytical session; precision and accuracy were quantified with blanks, replicated samples, as well as standard reference materials.

### Establishment of Diffusion Chambers/Microbial Traps (DC/MT)

A schematic representation of the diffusion chambers and microbial traps setup is provided in Supplementary Figure S1. Specifically, to setup the DC/MT, we used a sterile plastic plate with several even sized holes, 0.8 mm in height and 0.7 mm in diameter. We used 9 such holes forming a single DC or MT high throughput chamber (Jaswal et al., 2019a, b). This chamber was then sealed by sterilized 0.03 μm pore size polycarbonate membrane (GE Healthcare Biosciences, Pittsburgh, PA, United States) glued to one side of the tray (using silicone glue) in such a way that 9 holes on the tray would fall exactly toward the center of the membrane, to result in throughput cultivation (Supplementary Figure S1). To set up the DC chamber, 1 g soil was mixed with 9 ml of sterile normal saline and serially diluted to 10^–3^ dilution. One ml of this sample was then mixed with 9 ml of sterile molten agar at 45°C, to achieve a final concentration of 10^–4^. Approximately 435 μl of the 10^–4^ diluted soil sample which was mixed with agar inoculated into each of the 9 wells to completely fill the holes. After the agar solidified, a second 0.03 μm pore size membrane was glued on top to seal the chamber, thus establishing the diffusion chamber (DC). The 0.03 μm pore-size membrane permits diffusion of nutrients into the chamber thus facilitating the growth of environmental microbiota within the agar by mimicking *in situ* growth conditions with continuous, albeit diffusion limited exchange of nutrients and other growth factors such as cell-to-cell signaling molecules between the entrapped microbiota (Jaswal et al., 2019b).

To setup the microbial trap (MT), 435 μl of sterile molten agar at 45°C was added to each of the 9 wells in a plate similar to the DC chamber. After agar solidification, a 0.2 μm pore size sterile filter membrane was glued to seal the trap [for schematic, review Figure 1 from Jaswal et al. (2019b) and Supplementary Figure S1]. Note that serially diluted soils were not added into the MT agar chamber thus providing a surface for fungal hyphae to penetrate through the bottom membrane (0.2 μm) and colonize the chamber, and hence these are referred to as microbial traps. The DC and MT plates were then placed on the top of moist soils, as reported in previous studies (Jaswal et al., 2019a, b). These were incubated at 28°C for 20 days, and referred to as 1st generation (or Gen 1) of the DC/MT. The soils under the plates were mixed with a sterile spatula, every 2 days, to remove buildup of anaerobic conditions and homogenization of soil nutrients to feed the chambers. After 20 days, chambers were opened and the gel-embedded biomass was collected and homogenized twice by passaging through a 22-gauge needle syringe. This homogenized gel was divided into three different parts that were each used for DNA isolation, isolation of bacterial and fungal strains on Hg-supplemented Lysogeny Broth (LB) agar or Potato Dextrose Agar (PDA) respectively, and the remainder was used as inoculum for establishing the next DC/MT generation (Gen 2), which was further incubated for 20 days at 28°C, on moist Hg-contaminated soil. Soil mixing and flipping of the DC tray were performed similar to the incubation of Gen 1. Same procedure was performed at the end of Gen 2 and Gen 3 incubation periods, as was done at the end of Gen 1.

### Amplicon-Based Metagenomics

The DC/MT plugs were processed for extraction of genomic DNA using the DNeasy PowerLyzer Kit, according to the manufacturer’s instructions (Qiagen Inc., Germantown, MD, United States). DNA was extracted from each sample in duplicates and pooled prior to sequencing to minimize artifacts during the DNA extraction efficiencies. Both, the quantity and quality of the genomic DNA was evaluated using a micro-volume spectrophotometer (NanoDrop Technologies, Wilmington, DE, United States) and processed for 16S and ITS-based amplicon metagenomics, as shown before (Jaswal et al., 2019a, b). Sequence libraries were prepared using the Illumina Nextera XT kit, following the manufacturer’s instructions (Illumina Inc., San Diego, CA, United States), using 515F/926R primers for bacteria (Parada et al., 2016) and ITS1F/ITS2R for fungi, respectively (White et al., 1990). Sequencing was performed on an Illumina NextSeq500 instrument employing a mid-output kit with 2 × 150 paired-end sequencing.

The forward and reverse reads were merged prior to basic processing (sub-OTU) using PEAR (Yan et al., 2016). Reads were trimmed to delete the ambiguous nucleotides, primer sequences, based on the quality threshold of *p* = 0.01. Sequence reads that were devoid of primer sequence and any sequences less than 150 bp were discarded from further analysis. Chimeric sequences were also identified and removed using the USEARCH algorithm, as shown before (Edgar, 2010; Glöckner et al., 2017).

The obtained sequence reads were then analyzed using the standard QIIME pipeline with modifications to generate taxonomic summaries using sub-OTU resolution (Caporaso et al., 2010; Tikhonov et al., 2015). The resulting sequence files were merged with sample information with dereplication of sequences to produce a list of unique sequences for each sample. Sequences having an abundance of at least 10 counts were designated as seed sequences. USEARCH was then used to locate the nearest seed sequence for any non-seed sequence with a minimum identity threshold set at 98%. For any non-seed sequence matching a seed sequence, counts were merged with the seed sequence counts (Edgar, 2010). For any non-seed sequence not matching a seed sequence, it was retained as an independent sequence, respectively.

Using the above stated approach and modifications, taxonomic annotations were obtained for all the seed and unmatched non-seed sequences by using the USEARCH and Silva v132 (16S) or UNITE reference, keeping a minimum similarity threshold value of 90% (Edgar, 2010; Glöckner et al., 2017). Modifications in the analysis of metagenomic data was performed to improve annotation depth using the standard QIIME algorithm such that only those hits at each taxonomic level that had an assigned name were considered. For example, a reference sequence that was annotated as “k__Bacteria; p__Firmicutes; c__Clostridia; o__Clostridiales; f__Ruminococcaceae; g__; s__” was considered in the assignment of the taxonomic kingdom through family, but would not for the assignment of the genus or species. Furthermore, any hits in the reference database must have a minimum identity of 97 or 99% to be considered for genus or species level assignment, respectively. Taxonomic annotations and sequence abundance data were then merged into a single sequence table and used for plotting the data and further analysis.

### Nucleotide Sequence Accession Number

The 16S rDNA sequences of strains isolated in this study are deposited in NCBI GenBank, as shown in parentheses: S1 DC2 *Burkholderia* sp. strain SJN3 (MN936105), 3R1-1 *Burkholderia* sp. MBIC3837 (MN936104), 2B DC3 *Burkholderia* sp. 1 PSB-51 (MN936103), S3 DC1 *Burkholderia* sp. strain β-64 (MN936106), 3R1-3 *Coniochaeta velutina* (MN893457), 3B-5 *Rhodotorula pacifica* strain CBS 10070 (MN893459), 3R1-2 Fungal sp. 2 AKV-2015 (MN893460), 3S1-1 *Penicillium* sp. strain OUCMDZ-4754 (MN893461), 3S3-1 *Hypocrea nigricans* strain FD12 (MN893458), 3S3-5 *Rhodotorula mucilaginosa* isolate B3 (MN893462), H-02 DC3 *Mucor* sp. S1 (MN893463), and S1 MT6 *Penicillium* sp. isolate R57 (MN893464).

### Metagenomic Sequence Accession Numbers

The metagenomic 16S and ITS sequences obtained from this study are available from NCBI’s Sequence Read Archive/European Nucleotide Archive, accession # SRP211925, Bioproject # PRJNA550441.

### Differential Expression Analysis

Analysis of bacterial and fungal enhancement/depletion in reference relative to low, medium and high levels of THg contamination was performed using a differential expression approach. Specifically, differential expression statistics (fold-change and *p*-value) were computed using edgeR (Robinson et al., 2010; McCarthy et al., 2012), on raw expression counts obtained from metagenomic data after grouping the sequences based on their origination from Hg contamination levels, followed by a multi-group comparison analysis using the Generalized Linear Model functionality. *P*-values were adjusted for multiple testing using the false discovery rate (FDR) correction of Benjamini and Hochberg (Benjamini and Hochberg, 1995). Significant taxa were determined based on an FDR threshold of 5% (0.05).

### Heatmaps and Barplots

Hierarchical clustering of the log-scaled and *z*-scored normalized taxa abundance levels were performed, and data was plotted in a heatmap. Either the top 10 taxa were plotted (data not shown) or all taxa with average normalized abundance > 1%, as shown in stacked barplots.

### Microbiome Analysis

MicrobiomeAnalyst pipeline (Dhariwal et al., 2017) was run on sequence data processed as stated earlier, with the main intent to identify the core bacterial and fungal microbiomes along with diversity analysis. Data were filtered for low count and low variance based on prevalence (20% in all samples) and total sum scaling (TSS), which resulted in the removal of 540 low abundance features along with 53 low variance features based on inter-quantile range (iqr) for bacteria and 186 low abundance features and 5 low variance features based on iqr for fungi, respectively. Note that the “core” microbiome assignment refers to those taxa that were detected in a high fraction across the tested soils using the following threshold limits: sample prevalence (20%) and relative abundance of 0.01%, respectively. Dendrogram analysis was obtained at the genus level using the Bray–Curtis similarity matrix by selecting the experimental factor as grouped in the metadata file for all samples. Further ordination analysis on the amplicon-based metagenomics data was performed at the genus level using the MicrobiomeAnalyst pipeline, that included α-diversity (Chao1 measure with *T*-test/ANOVA), β-diversity plotted as PCoA (Bray–Curtis distance method with PERMANOVA), univariate analysis using *T*-test/ANOVA with an adjusted cutoff value of 0.05, and differential abundance analysis at the genus levels, calculated using EdgeR at an adjusted *p*-value cut off of 0.05.

### Functional Prediction Using PICRUSt2 Analysis

Functional metagenome was inferred from the OTU table using PICRUSt2 (Douglas et al., 2019). Higher level summaries of the predicted orthologous functions are created using KEGG pathway, module and BRITE hierarchical annotations (Kanehisa et al., 2017). Differential analyses of orthologous gene features were performed using the software package edgeR on raw sequence counts in a similar fashion as taxonomic summaries (McCarthy et al., 2012). Data were normalized as counts per million. Adjusted *p* values (*q* values) were calculated using the Benjamini–Hochberg false discovery rate (FDR) correction (Benjamini and Hochberg, 1995). Significant pathways were determined based on an FDR threshold of 5% (0.05).

### Isolation and Identification of Bacterial and Fungal Strains From DC and MT Chambers

Soil isolates were obtained from agarose plugs of gen 1, 2, and 3 by serially diluting the plugs with sterile water and spread plating onto LB agar media (for bacteria) and potato dextrose agar (PDA) media (for fungi), supplemented with mercuric chloride at a concentration of 5 μg/ml of Hg. Isolation of strains was performed as shown before (Jaswal et al., 2019a, b). DNA was extracted from the biomass that was colonizing the DC and MT gels as well as the isolated bacterial and fungal strains using DNeasy PowerLyzer Microbial Kit (Qiagen Inc., Germantown, MD, United States). Bacterial and fungal strains were taxonomically identified using the 16S and 18S rDNA sequence analysis. PCR was performed using 27F-1492R universal bacterial primers (Lane, 1991), using the following program: initial denaturing at 95°C for 3 min, followed by 35 cycles of denaturation at 94°C for 40 s, annealing at 55°C for 30 s, extension at 72°C for 60 s, followed by a final extension step of 72°C for 5 min. Fungal PCR was performed with FR1/NS1 universal fungal primers (Vainio and Hantula, 2000), with denaturing at 95°C for 8 min, followed by 35 cycles of denaturation at 95°C for 30 s, annealing at 47°C for 45 s, extension at 72°C for 60 s, and a final extension step of 72°C for 10 min. The sequences obtained were identified for taxonomy using the NCBI BLAST workflow.

### Evaluation of Mercury Resistance of the Isolated Strains

Isolated strains of bacteria and fungi were evaluated for their ability to resist mercury as follows. Mercuric chloride was added into LB growth media ranging from 0 to 5 mM. Overnight grown strains were added to this media at an initial OD_600_ of ∼0.3 and inoculated in Bioscreen C honeycomb plates (Growth Curves USA, NJ, United States), incubated at 30°C within the Bioscreen C system. All assays were conducted in triplicates and ensuing OD was measured every 4 h for 3 days at 28°C with constant shaking. Growth efficiencies of the isolated fungal strains on Hg were assessed by growing the strains on PDA in the presence of different concentrations of Hg (0–50 ppm). Two-day old PD broth grown fungal cultures were inoculated onto the center of each PD agar plates supplemented with Hg. The ensuing diameter of the fungal colonies was then measured every 24 h at 28°C for 7 days, and the increase in fungal biomass was used to represent Hg resistance.

### Statistical Analysis

The biogeochemical and metals data was processed as follows: triplicate runs were averaged to produce a single dataset representing each sample which was then imported into the Primer-E software suite (version 6.1.18; PRIMER-E, Ivybridge, United Kingdom). Normalization of data was performed using the log (X + 1) pre-treatment function and after transformation, a Bray–Curtis similarity matrix was generated and further analyzed by permutational multivariate analysis of variance (PERMANOVA). Data was also analyzed using a non-metric multidimensional scaling plot (NMDS) as well as canonical analysis of principal components (CAP). Dendrogram analysis was performed depicting each sample’s clustering pattern based on group average. Correlations between biogeochemical measurements and THg and MeHg were run using Microsoft excel package.

Canonical Correspondence Analysis (CCA) was also performed, which has been previously demonstrated to provide a meaningful constrained ordination of microbiological abundance data with environmental variables (Anderson and Willis, 2003), therefore, CCA was performed at different taxonomic levels to identify which of the tested environmental parameters likely shaped the microbial communities across variable soil contamination. Correlations of the soil measurements were evaluated between total mercury (THg), methylmercury (MeHg), total carbon (TC), total nitrogen (TN), and total phosphorus (TP) with the bacterial and fungal communities developed within the DC and MT chambers. CCA was performed using CANOCO v5 (Microcomputer Power Inc., Ithaca, NY, United States).

## Results and Discussion

### Mercury and Biogeochemical Measurements

Collected soils were analyzed for total mercury (THg), methylmercury (MeHg) along with biogeochemical parameters, to include total carbon (TN), total nitrogen (TN), total phosphorus (TP), extractable NH^+^_4_, extractable NO^–^_3_, extractable PO_4_^3–^ along with moisture content and loss on ignition, as summed up in Table 1. For mercury analysis, the mean percent recovery of TORT-3 (National Research Council of Canada; Ottawa, ON, Canada) was 102.1% (*SD* = 3.0%, *n* = 4), and method precision expressed as relative percent difference for replicated samples averaged 5.5% (*SD* = 5.2%, *n* = 8). The method detection limit was 0.002 ng for solid samples. For MeHg quantification, the mean percent recovery of BCR-580 (Joint Research Centre; Belgium) was 98.5% (*SD* = 3.2%, *n* = 4), and the relative percent difference for replicated samples averaged at 4.2% (*SD* = 0.9%, *n* = 4). The method detection limit was 0.02 ng/g for solid samples.

**FIGURE 2:**
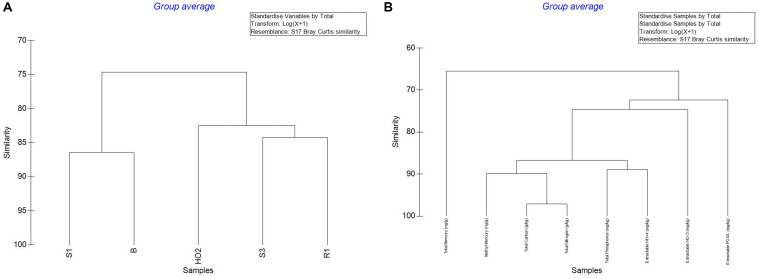
Cluster analysis of the measured parameters across the sampled sites to include THg, MeHg and soil biogeochemical measurements **(A)**; cluster analysis regardless of the sites chosen as the variable **(B)**.

Overall, analysis of Hg, MeHg and biogeochemical measurements in the SRS and ORR soils revealed that the following trends: concentration of TC was in the following order S1 > S3 > H-02-1 > R1 > B; TN also followed a similar trend except for site B which was slightly higher than site R1; TP concentrations also followed this trend except that the B site was relatively higher. LOI (loss on ignition) was several-fold higher in site H-02-1 relative to others. Extractable NH^+^_4_ was highest in sites S1 and B; extractable NO^–^_3_ was highest in H-02-1 and extractable PO_4_^3–^ was highest in S1 and H-02-1, respectively. THg levels were in the following order: B > S1 relative to other soils tested; MeHg levels were as follows: B > S1 > S3 with H-02-1 and R1 within the same range. As shown in Figure 2A, this binning scheme was statistically confirmed by cluster analysis using THg, MeHg and soil biogeochemical measurements which revealed the close similarity between sites B (high contamination) and S1 (medium contamination), respectively. Conversely, sites with lower levels of THg clustered separately, thus suggesting that the soil metal contamination potentially influenced the site characteristics. When cluster analysis was run regardless of the sites as variables, it was evident that MeHg clustered together with TC and TN but THg appeared separately as an outlier (Figure 2B), suggesting that MeHg formation is a function of soil carbon and nitrogen. Total phosphorus and extractable ammonia clustered together and so did extractable nitrate and phosphate. These observations are in line with previous studies in which microbially mediated MeHg production was linked to soil carbon, which potentially feeds the soil microbiota and enhance MeHg production. Furthermore, soil carbon also binds to THg, thus rendering it more bioavailable for Hg methylating microorganisms (Lambertsson and Nilsson, 2006). Furthermore, the TC and TN values were significantly correlated across all sites as expected and there was a significant correlation of extractable P with total P (data not shown). In relations to metal contaminants, soil THg concentrations analyzed ranged from 0.0098 to 1.68831 ppm and MeHg varied from 0.00027 to 0.00121 ppm, respectively (Table 1). A significant correlation (*R* = 0.83) across all sites with methylmercury and total mercury was also observed. For sites with relatively low total mercury, concentrations were methylmercury was 2.6% of total mercury while at the more contaminated sites (B and S1), methylmercury was 0.98% of total mercury (Table 1). This suggests that at higher total mercury concentrations, the methylation rate is comparatively slower which is likely related to suppression of microbial activity due to metal contamination. Overall, the soil biogeochemical and metal analyses resulted in the binning of locations being tested under the following levels of contamination: high (B), medium (S1), and low (S3, H-02), respectively. Contaminant levels in the reference site (R1) site were quite similar to sites binned under the low category, but for differential analysis conducted on the microbial communities to tease out the effects of contamination, site R was considered as reference, i.e., no history of direct contamination.

### Colonization of Bacterial and Fungal Communities Between Different DC/MT Generations

As stated before, application of culturomics has significantly improved cultivation rates of environmental microbiota which continues to broaden our understanding on the vast majority of soil microorganisms that remain uncultivable under standard laboratory growth conditions (Bodor et al., 2020). There are several strategies that can be employed to improve microbial growth and isolation from environmental samples and we have successfully applied the diffusion chamber (DC) and microbial trap (MT) techniques (Berdy et al., 2017) to understand uranium (U)-resistant bacterial and fungal assemblages in soils with long-term history of metal contamination (Jaswal et al., 2019a, b). Therefore, metagenomic analysis coupled with aforementioned DC/MT cultivation techniques, can serve as a sensitive and precise tool to assess the environmental microbiomes (bacterial) and mycobiomes (fungal) rendering their successful isolation and potential downstream applications, including bioremediation of metalliferous soil habitats.

**FIGURE 3:**
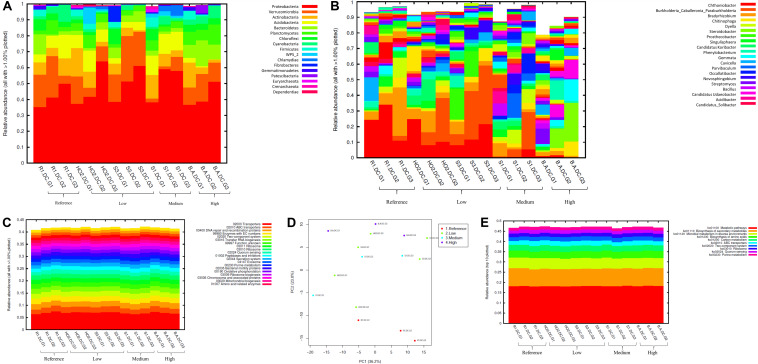
Bacterial diversity identified from the soils across different generations of the diffusion chambers (DC) plotted as relative abundances shown at the phyla **(A)**, and genus **(B)** level. Also shown are functional prediction analysis using PICRUSt plotted for BRITE level 3 (>1%) **(C)**; PCA analysis on BRITE level 3 data **(D)**; relative abundance of pathways identified **(E)**.

**FIGURE 4:**
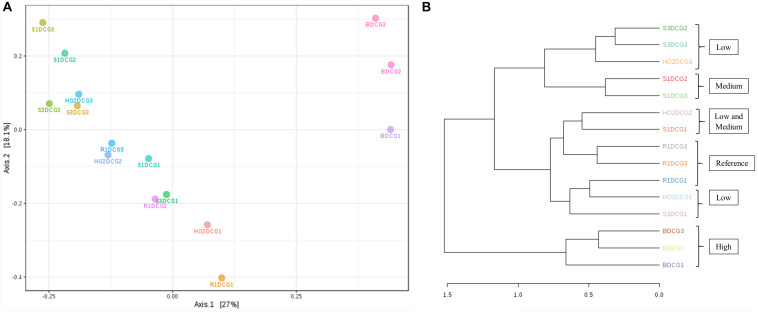
Shown are β-diversity and significance testing between the bacterial communities across different DC generations with ordination plotted as PCoA using the Bray–Curtis index **(A)**; dendrogram analysis of the bacterial communities across different DC generations using the Bray-Curtis index and the ward clustering algorithm **(B)**.

To further probe the Hg-cycling microbiota in sites containing variables levels of Hg contamination as stated in the previous section, i.e., high (B), medium (S1), low (S3, H-02), and reference site (R1) sites, amplicon-based sequencing was conducted to identify the bacterial and fungal assemblages that developed within the DC and MT chambers. Specifically, the sequence read counts from different generations of DC and MT were bioinformatically classified, as shown in Table 2. This revealed that a total of 556 Megabyte of sequences were obtained with 97–98% of the retrieved sequences from across the three different generations from low, medium, high levels of THg contamination as well as reference site, were taxonomically annotated to Domain Bacteria. Similarly, for the fungal Domain, a total of 139 Megabyte of sequences were obtained with 91–98% of the retrieved sequences annotated successfully. Utilizing this deep sequencing data, bacteria were taxonomically identified at the phylum and genera levels, across three generations of DC/MT established from 4 SRS and 1 ORR soils, as is shown in Figures 3, 4.

**TABLE 2:** Amplicon-based metagenomic data obtained from the reference, low, medium, and high contaminated soils used in this study.

BioSample	Experiment	SRA Study	Contamination Level	Sample	MBases	MBytes	Reads Annotated Taxonomically	Non-annotable Reads	Percent Passed Annotation
**(A) Diffusion Chamber (DC)**									
SAMN12136505	SRX6359646	SRP211925	Reference	R1-DC-G1	71	39	116759	1625	98.63%
SAMN12136488	SRX6359621	SRP211925	Reference	R1-DC-G2	66	36	108892	2117	98.09%
SAMN12136501	SRX6359545	SRP211925	Reference	R1-DC-G3	45	25	73616	2254	97.03%
SAMN12136582	SRX6359634	SRP211925	Low	S3-DC-G1	69	38	113265	2280	98.03%
SAMN12136496	SRX6359548	SRP211925	Low	S3-DC-G2	72	39	117517	2752	97.71%
SAMN12136509	SRX6359650	SRP211925	Low	S3-DC-G3	72	38	118664	1736	98.56%
SAMN12136538	SRX6359588	SRP211925	Low	HO2-DC-G1	72	39	116920	2693	97.75%
SAMN12136491	SRX6359618	SRP211925	Low	HO2-DC-G2	87	48	141119	4125	97.16%
SAMN12136504	SRX6359552	SRP211925	Low	HO2-DC-G3	68	36	111326	1962	98.27%
SAMN12136494	SRX6359613	SRP211925	Medium	S1-DC-G1	72	39	117709	1959	98.36%
SAMN12136487	SRX6359622	SRP211925	Medium	S1-DC-G2	54	30	88564	2044	97.74%
SAMN12136500	SRX6359544	SRP211925	Medium	S1-DC-G3	67	36	110227	1701	98.48%
SAMN12136484	SRX6359593	SRP211925	High	B-A-DC-G1	68	37	110871	2365	97.91%
SAMN12136497	SRX6359549	SRP211925	High	B-A-DC-G2	66	36	106932	2812	97.44%
SAMN12136510	SRX6359649	SRP211925	High	B-A-DC-G3	73	40	118492	3330	97.27%
**(B) Microbial Trap (MT)**									
SAMN12136517	SRX6359563	SRP211925	Reference	R1-MT-G1	18	10	30121	532	98.26%
SAMN12136530	SRX6359557	SRP211925	Reference	R1-MT-G2	18	10	29609	459	98.47%
SAMN12136543	SRX6359583	SRP211925	Reference	R1-MT-G3	18	10	30419	407	98.68%
SAMN12136521	SRX6359567	SRP211925	Low	S3-MT-G1	22	13	37311	555	98.53%
SAMN12136534	SRX6359561	SRP211925	Low	S3-MT-G2	15	9	25792	354	98.65%
SAMN12136547	SRX6359580	SRP211925	Low	S3-MT-G3	17	10	27381	2491	91.66%
SAMN12136519	SRX6359569	SRP211925	Low	HO2-MT-G1	16	9	26249	1204	95.61%
SAMN12136532	SRX6359559	SRP211925	Low	HO2-MT-G2	15	9	24990	701	97.27%
SAMN12136545	SRX6359582	SRP211925	Low	HO2-MT-G3	16	9	27134	666	97.60%
SAMN12136515	SRX6359565	SRP211925	Medium	S1-MT-G1	18	10	30227	368	98.80%
SAMN12136529	SRX6359558	SRP211925	Medium	S1-MT-G2	15	9	25873	357	98.64%
SAMN12136542	SRX6359592	SRP211925	Medium	S1-MT-G3	16	9	26787	415	98.47%
SAMN12136520	SRX6359570	SRP211925	High	B-A-MT-G1	13	8	22508	526	97.72%
SAMN12136533	SRX6359562	SRP211925	High	B-A-MT-G2	19	11	32555	429	98.70%
SAMN12136546	SRX6359581	SRP211925	High	B-A-MT-G3	5	3	9389	169	98.23%

*(A) Shows the bacterial sequence data and (B) shows the fungal sequence data, respectively. Samples prefixed with DC refer to diffusion chamber and MT to microbial traps; G1 through 3 refer to the different generations of DC and MT obtained from this study.*

Bacterial phylum level analysis revealed that 54.98% of read counts annotated with Proteobacteria followed by Verrucomicrobia (9.18%), Bacteroidetes (8.82%), Actinobacteria (5.95%), Acidobacteria (4.8%) as the top five phyla across all samples tested, regardless of the level of Hg contamination. A total of 20 phyla were present across the five tested soils, when plotted at the threshold of >1% relative abundance (Figure 3A). Noteworthy, a significant increase of proteobacterial lineages was observed within the DC chambers, as a function of generation times, such that the highest abundances of proteobacteria were observed in all the gen3 samples. This suggests that the DC approach facilitated conducive growth for the most abundant (core) phyla in the tested soils. The maximum enhancement of proteobacterial lineages were seen in sample H0-2, suggesting that these bacterial groups are primed in the H0-2 wetland soils to flourish under the DC growth conditions, such as nutrients and other bioactive compounds. Furthermore, as has been suggested before, it is very likely that proteobacterial lineages dominate in contaminated soils due to their ability to resist and even bioremediate heavy metals, hence the SRS and ORR metalliferous soils were also enriched in these lineages. This observation goes well with previous findings that have demonstrated proteobacteria as one of the dominant phyla in Hg-contaminated sediments in the ORR site (Vishnivetskaya et al., 2010). Similarly, other studies have also identified increased abundance of Proteobacteria as a function of Hg amendments (Ranjard et al., 2000; Rani et al., 2015; Mahbub et al., 2017). When levels of Hg contamination in the tested soils are given considerations, proteobacterial relative abundances steadily increased across gen1 through gen 3 in all the tested soils (B, R, H0-2, S3, and S1), respectively. Overall, these observations suggest that the DC technique resulted in enhanced colonization of proteobacterial members thus facilitating their subsequent isolation.

The predominant genera identified across the 5 soils were *Chthoniobacter*, *Burkholderia/Paraburkholderia* clade, and *Bradyrhizobium*, respectively (Figure 3B). The genus *Chthoniobacter* from phylum Verrucomicrobia have been documented from soil ecosystems with possible functions in the breakdown of organic carbon (Šrut et al., 2018). It is interesting to note that *Chthoniobacter* spp., were found to positively respond to high cadmium (Cd) treatments in earthworm gut samples (Šrut et al., 2018), and we speculate that this bacterial group also is likely engaged in Hg cycling in the tested soils. In soil B, which had the highest THg contamination, *Dyella* and *Chitinophaga* increased significantly across DC generations. Note that relative abundance of Chitinophagaceae were previously found to positively correlate to soil Hg in two recent studies (Liu et al., 2018, 2019). However, mechanism(s) by which *Chitinophaga* spp., resist or cycle Hg remain unknown at this time. *Dyella* spp., have also been found to strongly correlate with potential methylators in Hg contaminated soils (Niane et al., 2019). Hg resistant strains of *Dyella* have been isolated from the Hg contaminated gold mine sediments in Senegal (Mumtaz et al., 2013). Note that for all the soils, irrespective of the Hg levels, *Burkholderia/Paraburkholderia* spp., dominated the subsequent DC chambers. Using sequence analysis and multilocus sequence typing (MLST) conducted on *Burkholderia* spp., revealed their broad classification into two main groups; *Paraburkholderia* (mainly the environmental species) and the pathogenic forms (Oren and Garrity, 2015; Dobritsa and Samadpour, 2016). Note that several *Burkholderia* spp., have been previously shown to reduce Hg(II) to Hg(0) and/or degrade MeHg and it is clear that the DC approach enhanced their proliferation and successful isolation. It is noteworthy that *Burkholderia* spp., have also been previously identified in our ongoing projects focused on characterization of the soil bacterial diversity in metalliferous DOE soils (Jaswal et al., 2019a, b). In fact, other DOE metal contaminated environments that have undergone similar historical contamination exposures as the SRS and ORR sites, also contained elevated numbers of *Burkholderia* spp., such as the DOE Old Rifle Processing Site in Colorado (North et al., 2004; Moreels et al., 2008; Vishnivetskaya et al., 2010; Miller et al., 2013; Koribanics et al., 2015). This preponderance of *Burkholderia* spp., such as *B*. *xenovorans*, *B*. *vietnamiensis*, *B*. *fungorum*, *B*. *kururiensis*, *B*. *unamae*, *B*. *sartisoli*, and *B*. *phenoliruptrix*, have been widely utilized for bioremediation purposes due to their versatile biodegradative and metal resistance abilities (Coenye and Vandamme, 2003; Caballero-Mellado et al., 2004, 2007; O’Sullivan and Mahenthiralingam, 2005; Vanlaere et al., 2008; Zhang and Min, 2010; Ormeño-Orrillo et al., 2012; Suárez-Moreno et al., 2012; Oyetibo et al., 2013; Pérez-Pantoja et al., 2013; Koribanics et al., 2015; De Felice et al., 2016; Yang et al., 2016). However, fewer studies report on the use of *Burkholderia* spp., for treating heavy metals. Furthermore, invoking the criteria to bin microbiota as the core group(s), which refers to the set of taxa detected in a high fraction across the tested soils using the threshold levels stated elsewhere in this work, it was clearly shown that *Burkholderia*, *Bradyrhizobium* and *Chthoniobacter* spp. predominated across all soils tested (Supplementary Figure S2), similar to the data shown in Figure 3B. This established the ubiquity of these bacterial groups in the tested soils with variable levels of Hg impact. Furthermore, the propensity of the identified predominant or core microbiomes relative to the levels of contamination were established by differential analysis, as shown in Supplementary Figure S3. Among the suite of bacterial taxa identified in the samples, the core communities that included *Burkholderia*, *Bradyrhizobium*, and *Chthoniobacter* spp. were enriched as a function of soil contamination levels such that the abundance of these taxa were several-fold higher, as indicated by red arrows in Supplementary Figure S3, in the soils with highest (B) and medium (S1) levels of contamination. Interestingly, there were several other taxa that also were significantly enriched only in the highest contaminated soils, as indicated by differential analysis where the low, medium and high levels of soil THg contamination was statistically evaluated relative to the reference soils; the taxa found at higher abundances are indicated by parenthesis and red arrows in Supplementary Figure S3. Surprisingly, these did not include any of the 9 Hg resistant genera as reported by Rani et al. (2015), but did include a plethora of other bacterial genera that are well-known to resist uranium such as *Rhodanobacter* and *Caulobacter*, respectively (Kostka et al., 2012; Chung et al., 2014; Park and Taffet, 2019). It is highly likely that these genera possess ecologically beneficial traits that facilitate their survival under the tested metalliferous soils and further research on these bacterial genera is recommended for environmental remediation and development of biosensors.

How are microbially mediated functions affected as the bacterial taxa shift between different generations of DC? This is a central question that remains unaddressed in many previous studies based on assessment and isolation of bacterial taxa using the diffusion chamber approach. To address this, PICRUSt2 analysis was conducted on the amplicon metagenomics data to infer functional and metabolic responses of bacterial and fungal communities that developed across three different generations of DC/MT in our study. PICRUSt2 is based on the KEGG database, which is an integrated resource comprising of 15 manually curated databases under the following information categories: PATHWAY, BRITE and MODULE. These analyses garnered significant insights into microbial cellular and metabolic functions in the tested soils under DC and MT conditions. The top categories on the BRITE level 3 bacterial-based analysis revealed transporters to be predominantly active in the DC experiment (Figure 3C) and these transport functions increased, albeit slightly across the three different DC generations, indicating that microbial communities were metabolically actively and likely use transport function to survive in metalliferous soils. PCA analysis revealed separate clustering of reference G2 and G3 relative to other samples (Figure 3D). Moreover, for the most part, G2 and G3 samples, regardless of their contamination levels, clustered together indicating that the DC condition facilitated microbial functioning. Furthermore, metabolic pathways, biosynthesis of secondary metabolites, metabolism in diverse environments and transport predominated when top 10 bacterially mediated pathways were plotted (Figure 3E).

To further evaluate the statistical differences between microbiomes enriched in different generations of the DC/MT, β-diversity at the genus levels were estimated and ordination plotted as PCoA using the Bray–Curtis index (Figure 4A). β-diversity in context to this study is the similarity measure of microbial diversity between multiple communities and/or soil samples, which revealed close clustering of the bacterial diversity identified from the highest contaminated soil B, across three different generations from DC relative to the medium and low contaminated soils. Dendrogram analysis further established that regardless of the DC generations, tight clustering was observed across all tested soils as a function of their contamination levels, indicating that soil contamination could have shaped the nature of microorganisms that could survive in the tested soils (Figure 4B).

**FIGURE 5:**
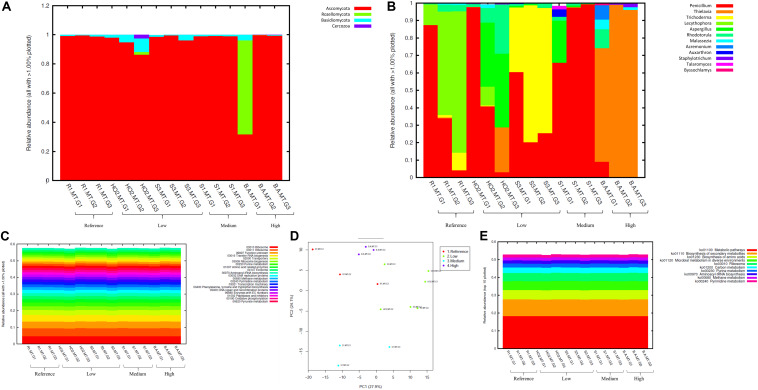
Fungal diversity identified from the soils across different generations of the microbial traps (MT) plotted as relative abundances shown at the phyla **(A)**, and genus **(B)** level. Also shown are functional prediction analysis using PICRUSt plotted for BRITE level 3 (> 1%) **(C)**; PCA analysis on BRITE level 3 data **(D)**; relative abundance of pathways identified **(E)**.

In addition to the evaluation of bacterial communities from the DC approach, we also investigated the developing mycobiomes, i.e., the fungal communities in the MT chambers established using Hg contaminated soils. It is noteworthy that despite the ability of fungal communities to withstand higher concentrations of environmental contaminants and even outcompete bacterial communities, they continue to remain ignored and therefore, understudied in environmental microbiome surveys and applications (Rajapaksha et al., 2004; Maddela et al., 2015; Agarwal et al., 2018; Oladipo et al., 2018). In fact, our previous metagenomic studies coupled with the DC/MT technique conducted on uraniferous soils, revealed the predominance of Ascomycota phylum, with *Penicillium* as the most dominant fungal genus (Hemme et al., 2015; Feng et al., 2018; Jaswal et al., 2019b). Isolation and genomic analyses on the retrieved fungal isolates have also shed light on a suite of environmentally, ecologically and evolutionary beneficial traits, such as heavy metal resistant genes, membrane transport, efflux, as demonstrated for other metal contaminated ecosystems (Leitão, 2009; Hemme et al., 2015; Chauhan et al., 2018; Feng et al., 2018). Therefore, when metagenomics is combined with the microbial trap technique, it can provide a deeper understanding on “who is there” and “what are they doing” in context to the microbial ecology of the legacy contaminated soils. Specifically, our studies on uraniferous soils confirmed that the chambers facilitated proliferation and subsequent isolation of specific microbiota with environmentally relevant functions. With the MT technique optimized for metalliferous soils, we evaluated the five mercury contaminated soils in which the predominant bacterial communities have already been characterized, as stated earlier in this manuscript. The ITS metagenomic analysis for fungal communities colonizing across gen 1–3 experiments are presented in Figure 5. In line with our previous studies, the Ascomycota phylum dominated in all soil samples tested, regardless of their contamination levels or biogeochemical status (Figure 5A). Note that Ascomycota has also been observed to be present in Hg contaminated soils from the former mercury mining plant in Rudňany in central Slovakia (Urík et al., 2014) as well as another study on different heavy metal contaminants (Lin et al., 2019). Moreover, Ascomycota fungal species are saprophytic and serve as one of the main decomposers in soil with unique ability to recycle even refractory organic matter, that includes lignin and keratin, thus serving as key players in nutrient cycling. It can be hypothesized that Ascomycota possesses stronger environmental adaptability which enabled this phylum to be so ubiquitous in the tested soils. Because Ascomycota has the propensity to decompose both plant and animal residue, it is a critical player in the recycling of unusable environmental substances making it available to other biota, such as bacterial assemblages.

At the genus level *Penicillium*, *Thielavia*, and *Trichoderma* dominated the MT chambers (Figure 5B). Note that the *Penicillium* genus, belonging to the Ascomycota phylum, have been shown to resist metals and be agents of environmental detoxification associated with metals (Nazaret et al., 2003; Harms et al., 2011). In the highest Hg contaminated soil (B), *Thielavia* spp. became highly abundant going from gen1 to gen 3, which indicates the ability for this fungus to thrive in the tested conditions and resist contaminants that leach into the chamber from the moist soil placed below the chambers. Not much is known on the ability of *Thielavia* spp. for Hg bioremediation and it would be of significant interest to isolate these fungal members and evaluate their spectrum of bioremediation potential. Furthermore, invoking the criteria to bin the fungal mycobiomes as the core group(s), which refers to the set of taxa detected in a high fraction across the tested soils using the threshold levels stated elsewhere in this work, it was clearly shown that *Penicillium* spp. predominated across all soils tested (Supplementary Figure S4), similar to the data shown in Figure 5B. This established the ubiquity of these fungal genera in the tested soils with variable levels of Hg impact. These observations were statistically validated by conducting differential analysis, which verified the previous observations such that the fungal genera *Penicillium*, *Thielavia*, *Trichoderma*, and *Aspergillus* were significantly abundant in the highest contaminated soils (B), as indicated by red arrows in Supplementary Figure S5. Overall, it can be concluded that these fungal genera have evolved to recruit ecologically beneficial traits that ensures their survival under the tested metalliferous soils. Also noteworthy is to mention that our previous MT experiments resulted in the isolation of a novel *Penicillium* sp. MT2, as indicated by comparative genomics and bioinformatics (Jaswal et al., 2019b). Specifically, this strain possessed 1904 genes unique genes (16.5% of the total genome), relative to four closest relatives, with many genes with functions related to heavy metal and drug resistance and a variety of efflux pumps. Taken together, this is strong evidence that *Penicillium* spp., have recruited genome-enabled functions for resistance and bioremediation to survive in their native SRS and ORR soil habitat contaminated with heavy metals including uranium and mercury.

Prediction of functions on fungal metagenomic communities were also conducted in the same way as was done for bacterial communities using PICRUSt, which showed ribosome and unknown function as the top categories (Figure 5C) along with transport and these functions did not vary across different generations. PCA analysis revealed closer association of all generations from the most contaminated soil (B) relative to other samples; moreover, clustering was mostly between samples with similar contamination levels but not generations (Figure 5D). Similar to bacterial analysis, metabolic pathways and biosynthesis of secondary metabolites as well as metabolism in diverse environments were among the top 10 fungal-mediated processes (Figure 5E).

**FIGURE 6:**
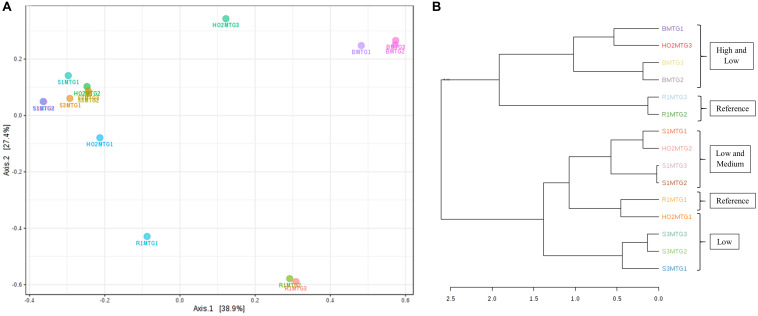
Shown are β-diversity analysis and significance testing between the fungal communities across different MT generations with ordination plotted as PCoA using the Bray–Curtis index **(A)**; dendrogram analysis at the fungal communities across different MT generations using the Bray-Curtis index and the ward clustering algorithm **(B)**.

Metagenomic data on soil bacterial and fungal communities originating from variable levels of Hg-contamination and developing within the DC/MT chambers were then evaluated using multivariate statistical analyses. Similar to the bacterial analysis, β-diversity was estimated at the genus levels and PCoA ordination plots were generated using the Bray–Curtis index (Figure 6). As was observed for the bacterial beta diversity analysis, all the three generations of MT for the highest contaminated site (B) clustered close together and for the most part, the medium and low contaminated sites clustered together on a separate axis (Figure 6A). Dendrogram analysis also confirmed that the soil contamination level exhibited a tighter control on the MT fungal communities relative to the three different generations/incubation times (Figure 6B).

### Statistical Evaluation of Bacterial and Fungal Communities With Site Environmental Measurements

**FIGURE 7:**
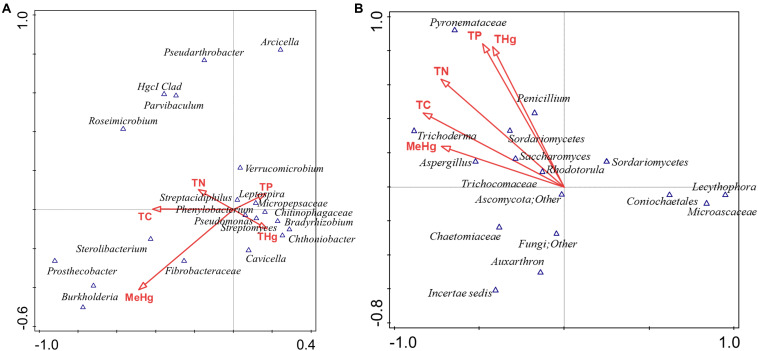
Canonical correspondence analysis (CCA) to evaluate the correlations between the effect of various environmental, biogeochemical and the microbial diversity. The first two axes of CCA1 and CCA2 shown represent the relationships of environmental variables with bacterial diversity in DC **(A)** and fungal diversity in MT **(B)**.

To further test which of the measured environmental factors shaped the bacterial and fungal diversity in the Hg-impacted soils, CCA analysis was carried out between the top five bacterial and fungal genera, which is presented in Figure 7. This confirmed that total mercury (THg) likely shaped the predominant genera identified in previous analysis, i.e., *Chthoniobacter*/*Bradyrhizobium* spp., correlated to THg whereas *Burkholderia* spp., correlated with MeHg with TC, TN, and TP correlating to other less abundant microbiota (Figure 7A). CCA analysis of the major fungal genera identified from the MT approach are presented in Figure 7B, which revealed that among the evaluated environmental measurements, THg and MeHg grouped together as having a cumulative effect on the fungal diversity such that *Penicillium* spp., correlating with THg whereas *Trichoderma* spp., and *Aspergillus* spp., correlated with MeHg, respectively. Overall, this analysis suggests that the predominant bacterial and fungal communities identified in this, as well as our previous studies in the DOE contaminated soils are shaped by heavy metals, such as mercury, and not so much by the differences in the soil biogeochemistry. These are critical findings which provided the rationale to isolate the statistically relevant microbiota and evaluate their propensity to resist Hg.

### Isolation of Bacteria and Fungi From the DC/MT Chambers

To confirm that the predominant bacterial and fungal communities are also represented in cultivation-based analysis, we performed isolation experiments from the DC/MT agar plugs onto growth media supplemented with Hg so that the soil isolates can be evaluated for environmental applications. Specifically, the isolations were performed from generation 3 of the DC and MT plugs by dilution and spread-plating onto LB/PD agar supplemented with 5 ppm Hg. From the DC approach, four bacterial strains (called as S1 DC2, 3R1-1, 2B DC3, and S3 DC1), and three fungal strains (called as H-02 DC3, 3B-5, and 3S3-1) were isolated. Using the MT approach, five fungal strains (called as 3S1-1, 3R1-2, 3S3-5, S1 MT6, and 3R1-3) were isolated. Noteworthy is that all isolated bacteria belong to *Burkholderia* genus, which were also the main bacterial genera identified in the tested soils using metagenomics (Figure 3B). Conversely, the isolated fungal strains were more diverse and were identified as *Penicillium*, *Rhodotorula*, *Hypocrea*, *Mucor*, and *Coniochaeta*. These isolates were further screened for their resistance to Hg as stated in the next section.

### Screening of the Isolated Strains for Mercury Resistance

**FIGURE 8:**
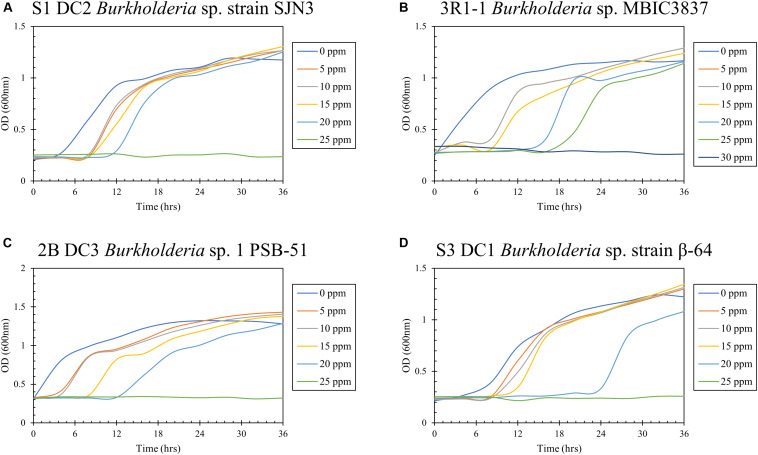
Determination of mercury resistance of bacterial strains **(A)** S1 DC2 *Burkholderia* sp. strain SJN3; **(B)** 3R1-1 *Burkholderia* sp. MBIC3837; **(C)** 2B DC3 *Burkholderia* sp. 1 PSB-51; and **(D)** S3 DC1 *Burkholderia* sp. strain β-64, isolated from DC by measuring OD (600 nm) in LB supplemented with variable Hg concentrations.

**FIGURE 9:**
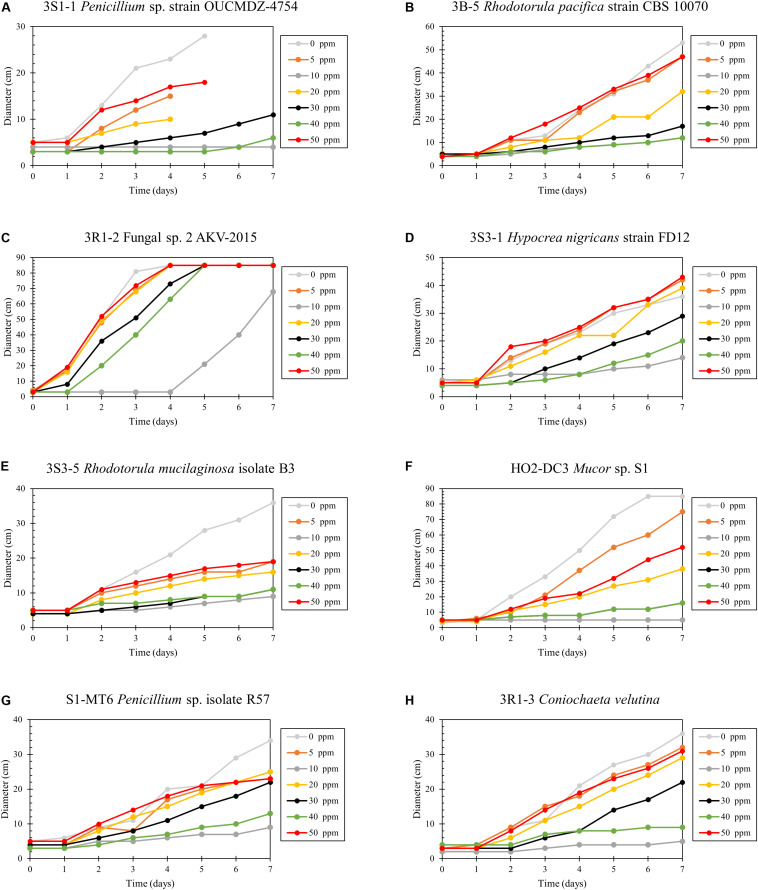
Determination of mercury resistance of fungal strains **(A)** 3S1-1 *Penicillium* sp. strain OUCMDZ-4754; **(B)** 3B-5 *Rhodotorula pacifica* strain CBS 10070; **(C)** 3R1-2 Fungal sp. 2 AKV-2015; **(D)** 3S3-1 *Hypocrea nigricans* strain FD12; **(E)** 3S3-5 *Rhodotorula mucilaginosa* isolate B3; **(F)** HO2-DC3 *Mucor* sp. S1; **(G)** S1-MT6 *Penicillium* sp. isolate R57; and **(H)** 3R1-3 *Coniochaeta velutina*, isolated from MT by measuring diameter of growth on PD agar supplemented with variable Hg concentrations.

Hg resistance in bacterial strains was evaluated by measuring bacterial cell growth (OD_600_) in LB media supplemented with Hg. Note that all four of the isolated bacterial strains in this study grew at Hg concentrations of 20 ppm (Figure 8) and by inference, were considered to be Hg resistant. Of all these strains, 3R1-1 was able to show slight growth at 25 ppm Hg (Figure 8B). The fungal strains were grown in PDA supplemented with 50 ppm Hg and their resistance was measured as an increase in colony diameter over time (Figure 9). All the eight isolated fungal strains from DC and MT treatments exhibited good growth at the highest tested Hg concentration (50 ppm) (Figure 9C), indicating that the isolated strains are resistant to high concentrations of Hg; typical resistance to Hg in previous reports have been found in the range of 10–30 ppm (Chasanah et al., 2018). On some concentrations with strain 3S1-1, contamination appeared after day 4, at which point these experiments were stopped, which explains the missing data points. Moreover, some strains showed growth at higher concentrations of Hg but their colony diameters were inhibited at lower concentrations, which is most likely due to our inability to start the assay with the same amount of the fungal biomass used as the starting material. Regardless of this, the isolated strains appear to be far more resistant to Hg than the concentration showed in the tested soils- THg concentrations varied from 0.0098 to 1.68831 ppm and MeHg varied from 0.00027 to 0.00121 ppm, respectively (Table 1). Several fungal strains isolated from a mercury mining plant in Rudňany in Slovakia were also shown to resist up to 32 ppm Hg (Urík et al., 2014). It should also be noted a standardized assay to evaluate mercury resistance across different bacterial and fungal strains is lacking which makes it difficult to compare HgR across strains using the minimal inhibitory concentration protocol (de Luca Rebello et al., 2013). Regardless, it can be concluded that all the isolated bacterial and fungal strains in this study exhibited significant resistance to Hg and are likely key Hg-cycling microbial players in the SRS and ORR soil habitats.

## Conclusion

The use of meta-omics studies are enhancing our understanding and appreciation of the myriad of ecosystem services rendered by environmental bacterial and fungal communities (Marco and Abram, 2019). Ecosystem services are broadly defined as benefits obtained by the society and toward this end, microbially based services provide several benefits to sustain our planet, such as support production of food and enzymes for industrial processes; maintenance of water quality and reducing contamination as well as plant growth promoting effects and biogeochemical cycling of nutrients. One critical role that underpins ecosystem services rendered by environmental microbiota includes their broad bioremediative potential, for example against the widespread heavy metals present in the environment. Toward this direction, to our knowledge, this is the first metagenomics-based assessment and isolation of bacterial and fungal assemblages that colonized in diffusion chambers and microbial traps established with long-term heavy metal (mainly mercury and uranium) contaminated soils. We believe that this approach can be a cost-effective approach to assess microbially driven ecosystem service functions in a variety of environmental ecosystems. Because the findings presented herein were obtained from soils collected only at one time point, any seasonal variations remain unclear, thus, results should be interpreted cautiously given this limitation. Furthermore, because DC chambers are established with molten agar at 45°C, it is also likely that heat-sensitive microorganisms may become impacted and remain underrepresented using this technique. Moreover, the DC/MT approach will likely target only those microorganisms that are amenable to cultivation under the DC/MT conditions and hence is potentially a biased assessment of the microbial diversity in its entirety, as can be provided using metaomics techniques, e.g., metatranscriptomics or metaproteomics assessment of Hg-cycling processes (Christensen et al., 2019). Regardless of this limitation, it can be concluded that DC and MT techniques do facilitate recovery and downstream studies on Hg-resistant microbiota, along with enhancing our understanding of bacterial and fungal communities that persist in legacy contaminated soils. Because DOE sites have complex contamination issues, it is critical to tease out the linkages between bacterial and fungal communities and their functional relevance in context to heavy metal cycling for better stewardship and restoration of such historically contaminated ecosystems. Toward this direction, metagenomics coupled with DC/MT techniques can be an extremely powerful tool to probe “who is there” and “what are they doing” relative to the microbial ecology of legacy contaminated sites in the US.

## Data Availability Statement

The datasets generated for this study can be found in the 16S rDNA sequences of strains isolated in this study are deposited in NCBI GenBank, as shown in parentheses: S1 DC2 *Burkholderia* sp. strain SJN3 (MN936105), 3R1-1 *Burkholderia* sp. MBIC3837 (MN936104), 2B DC3 *Burkholderia* sp. 1 PSB-51 (MN936103), S3 DC1 *Burkholderia* sp. strain β-64 (MN936106), 3R1-3 *Coniochaeta velutina* (MN893457), 3B-5 *Rhodotorula pacifica* strain CBS 10070 (MN893459), 3R1-2 Fungal sp. 2 AKV-2015 (MN893460), 3S1-1 *Penicillium* sp. strain OUCMDZ-4754 (MN893461), 3S3-1 *Hypocrea nigricans* strain FD12 (MN893458), 3S3-5 *Rhodotorula mucilaginosa* isolate B3 (MN893462), H-02 DC3 *Mucor* sp. S1 (MN893463), and S1 MT6 *Penicillium* sp. isolate R57 (MN893464), respectively. The metagenomic 16S and ITS sequences obtained from this study are available from NCBI’s Sequence Read Archive/European Nucleotide Archive, accession # SRP211925, Bioproject # PRJNA550441.

## Author Contributions

RJ, MA, BE, JH, and RR established the chambers. RJ isolated the strains and performed Hg resistance assays. AP analyzed the metagenomic samples and conducted the bioinformatic analysis. RJ, AP, and AC performed the statistical analysis. JW performed the biogeochemical experiments. XX analyzed THg and MeHg concentrations. RJ, AP, SB, XX, JW, and AC wrote the manuscript.

## Conflict of Interest

The authors declare that the research was conducted in the absence of any commercial or financial relationships that could be construed as a potential conflict of interest.

## Supplementary Material

FIGURE S1 Schematic outline of the diffusion chambers and microbial traps established in the study on soils containing low, medium, and high levels of mercury as well as a reference soil that did not have any direct exposure to Hg.

FIGURE S2 Bacterial groups identified as the core microbiome in the metagenomic libraries from the soils tested in this study.

FIGURE S3 Differential analysis on bacterial communities obtained from reference samples relative to low, medium, and high levels of mercury contamination. Genera that are differentially abundant relative to the levels of total mercury (THg) contamination are shown in parenthesis and/or red arrows.

FIGURE S4 Fungal groups identified as the core mycobiome in the metagenomic libraries from the tested soils.

FIGURE S5 Differential analysis on fungal communities obtained from reference samples relative to low, medium, and high levels of mercury contamination. Genera that are differentially abundant relative to the levels of total mercury (THg) contamination are shown in parenthesis and/or red arrows.
