# Supplementary figures and images for: Characterization of Bacterial and Fungal Assemblages From Historically Contaminated Metalliferous Soils Using Metagenomics Coupled With Diffusion Chambers and Microbial Traps
Source: Front Microbiol. 2020 Jun 10;11:1024. doi: 10.3389/fmicb.2020.01024 (PMC7325934; doi:10.3389/fmicb.2020.01024)

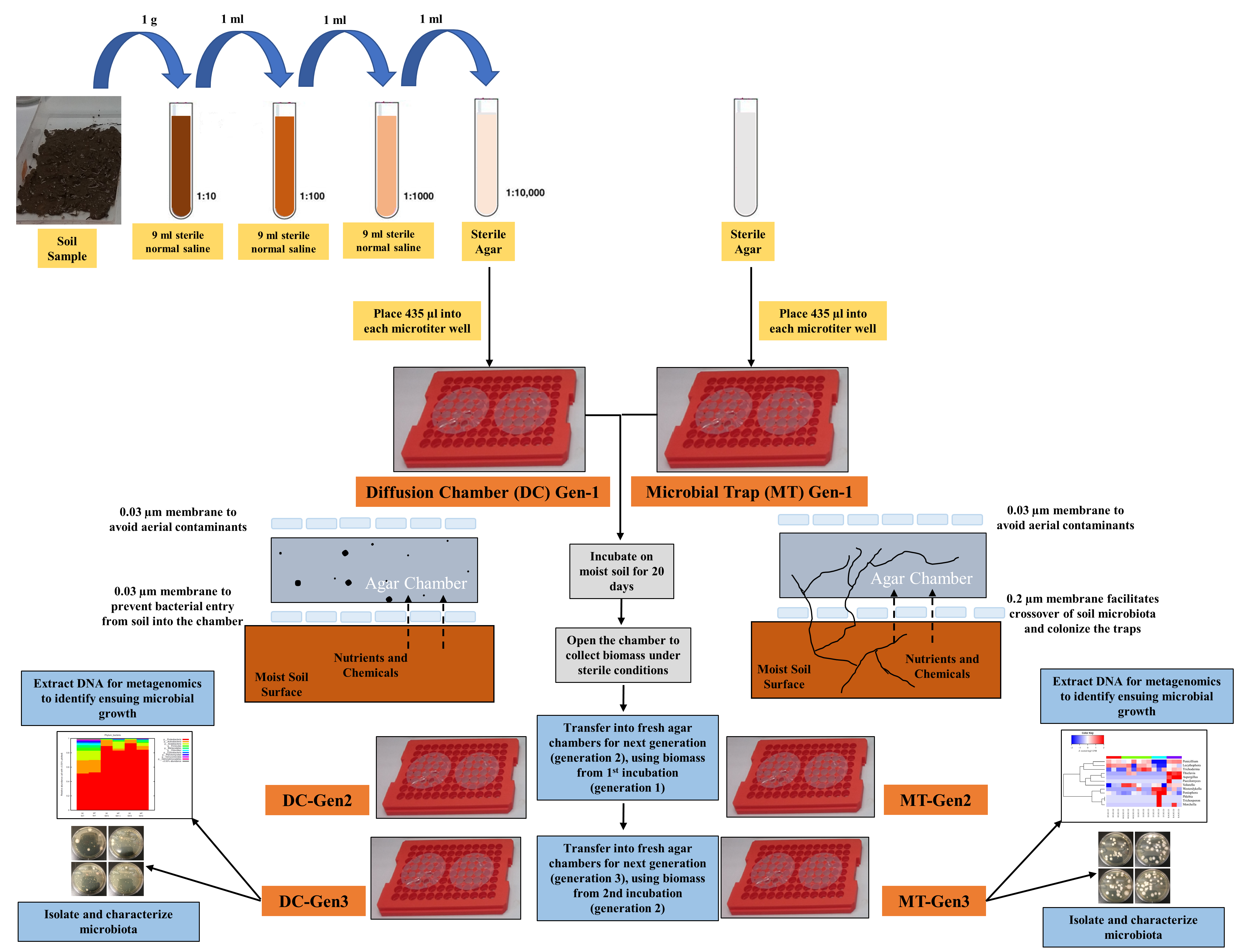

Supplement: FIGURE S1 — Schematic outline of the diffusion chambers and microbial traps established in the study on soils containing low, medium, and high levels of mercury as well as a reference soil that did not have any direct exposure to Hg. [file Image_1.tif]

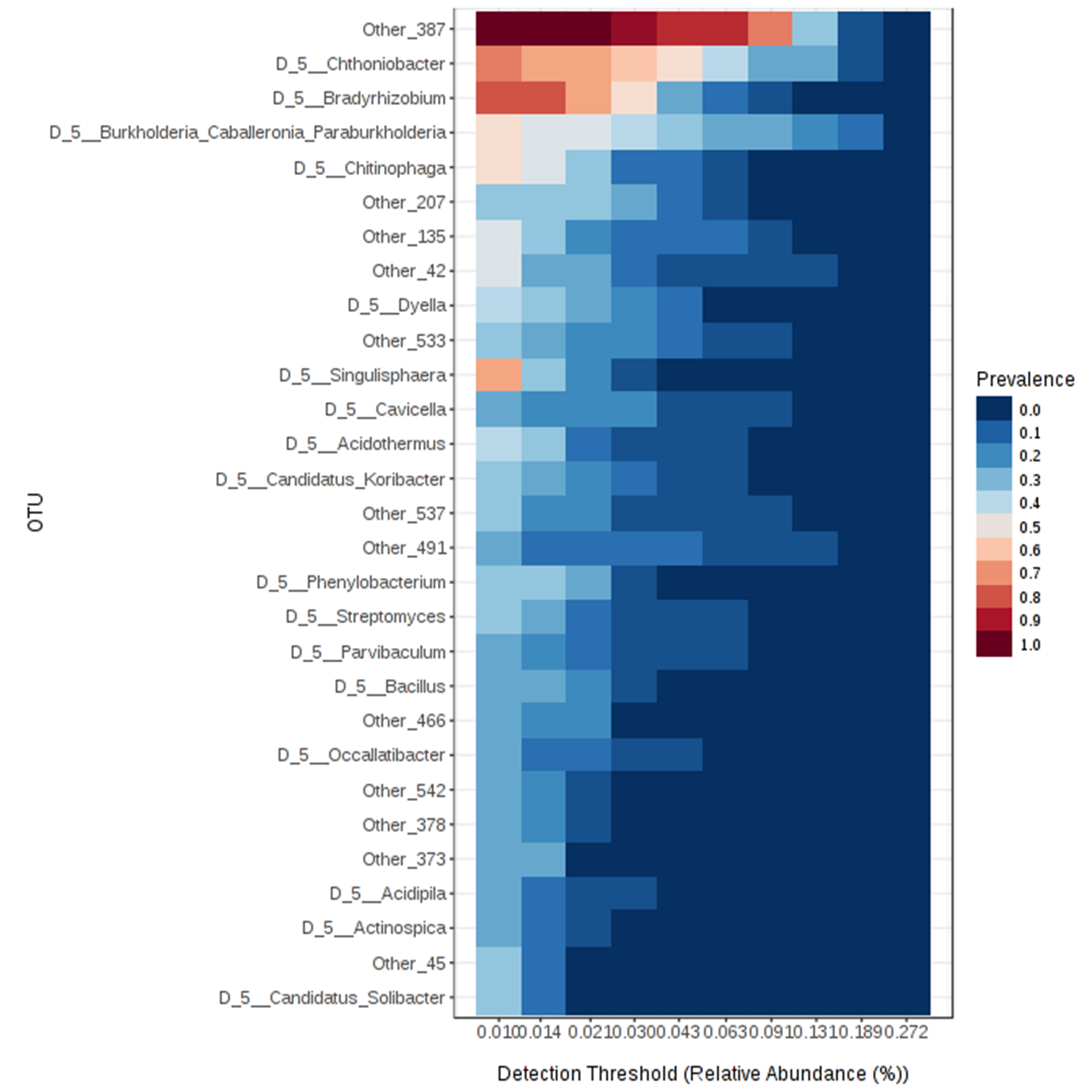

Supplement: FIGURE S2 — Bacterial groups identified as the core microbiome in the metagenomic libraries from the soils tested in this study. [file Image_2.tif]

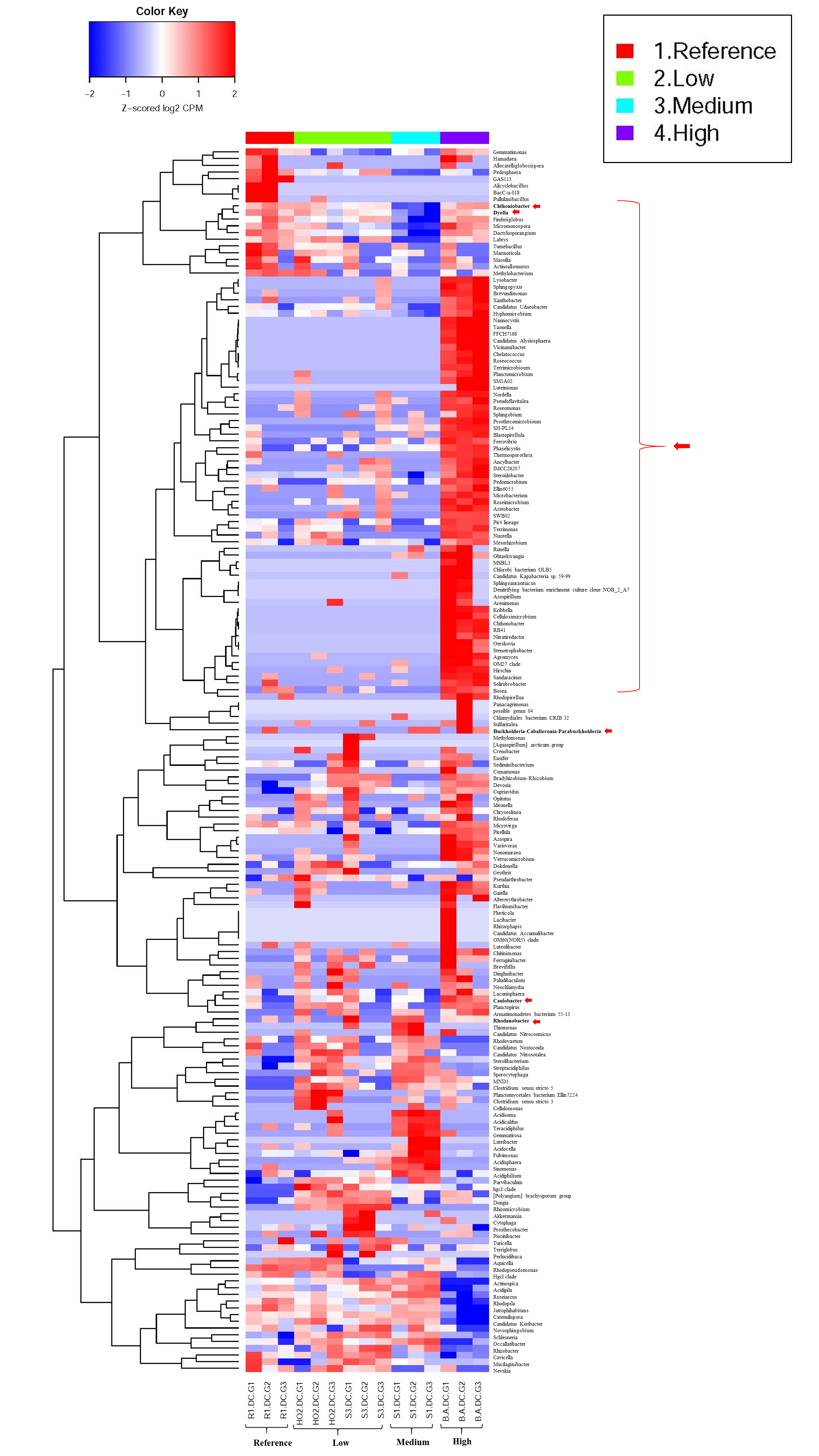

Supplement: FIGURE S3 — Differential analysis on bacterial communities obtained from reference samples relative to low, medium, and high levels of mercury contamination. Genera that are differentially abundant relative to the levels of total mercury (THg) contamination are shown in parenthesis and/or red arrows. [file Image_3.tif]

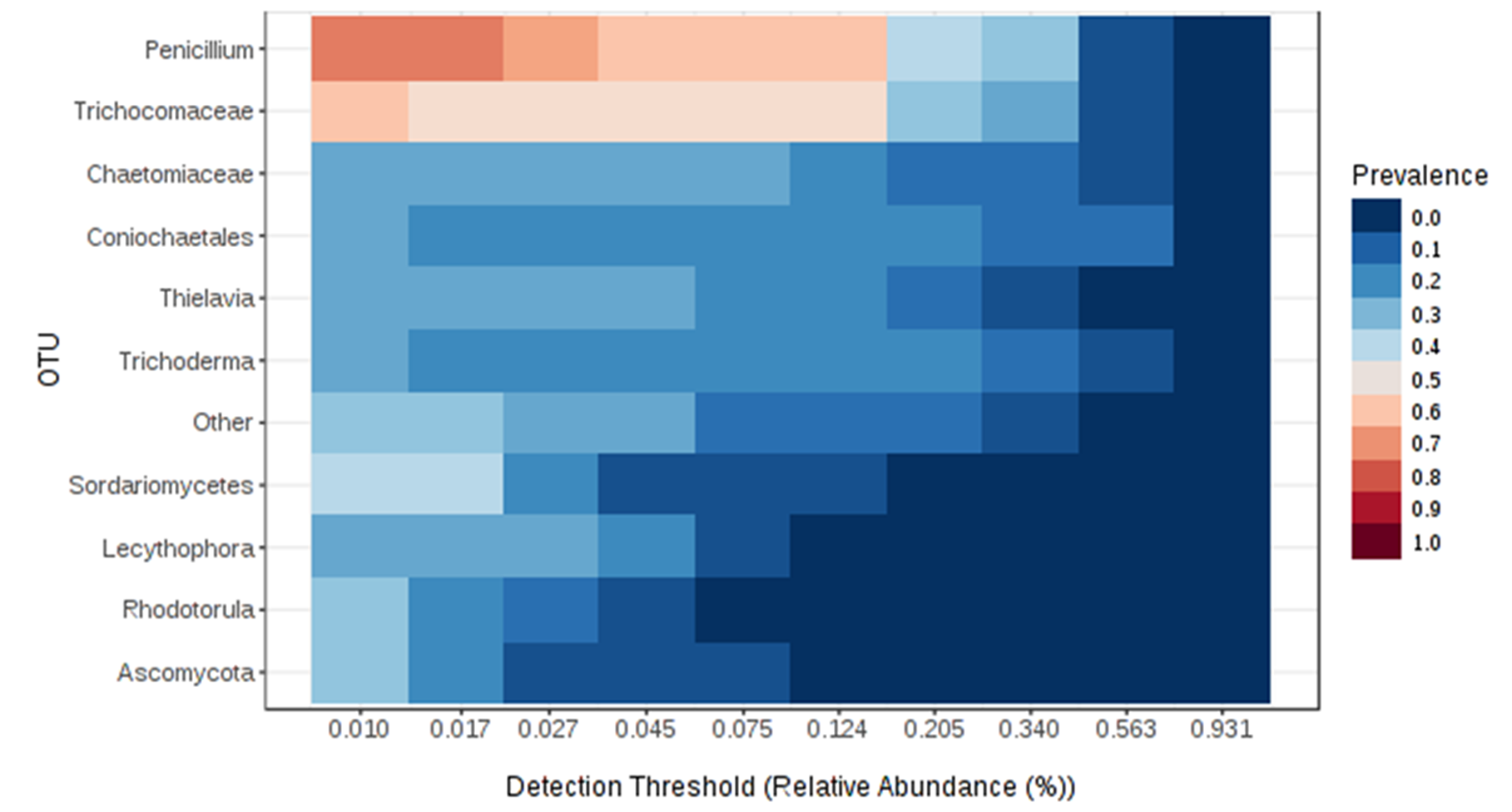

Supplement: FIGURE S4 — Fungal groups identified as the core mycobiome in the metagenomic libraries from the tested soils. [file Image_4.tif]

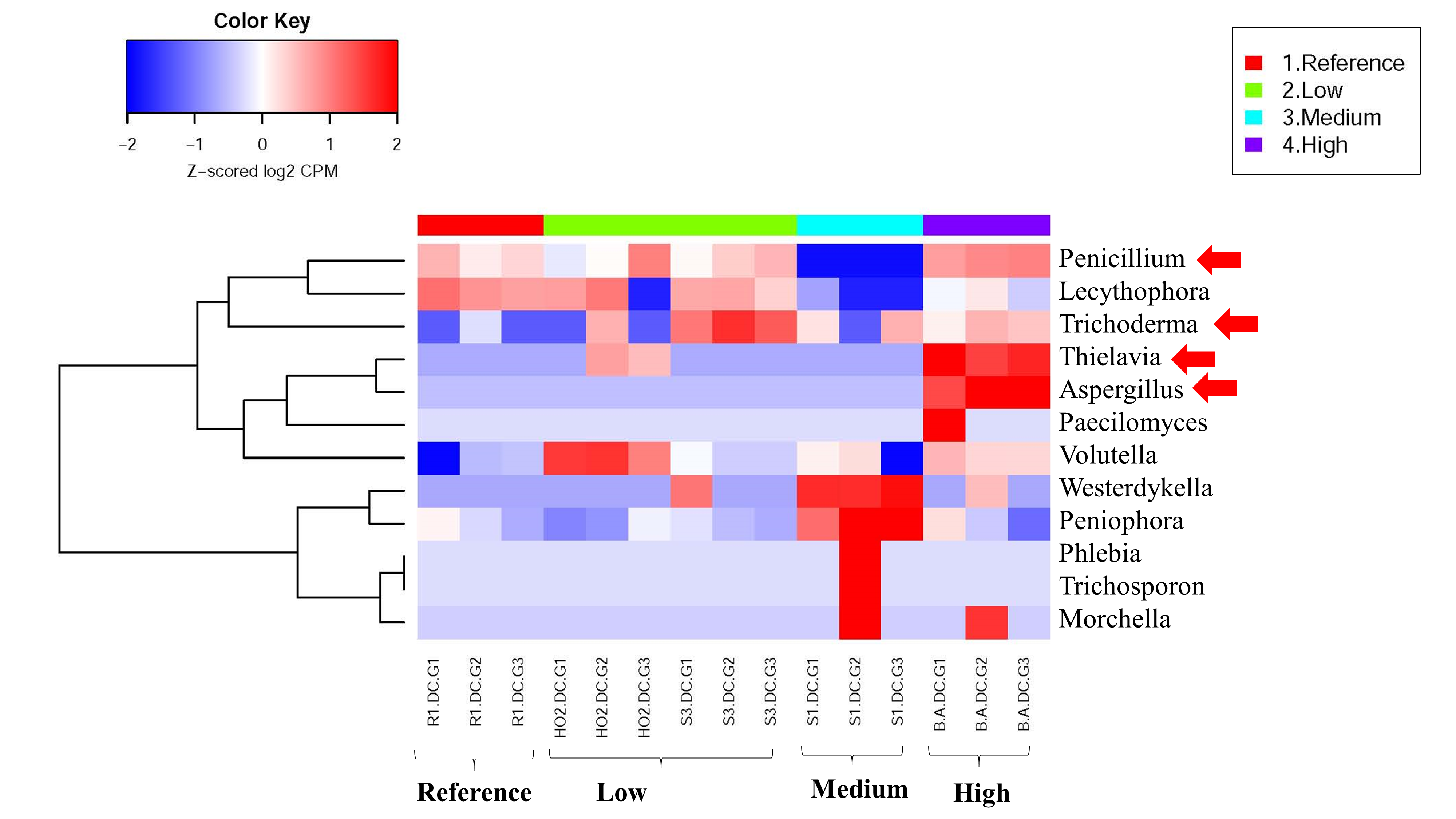

Supplement: FIGURE S5 — Differential analysis on fungal communities obtained from reference samples relative to low, medium, and high levels of mercury contamination. Genera that are differentially abundant relative to the levels of total mercury (THg) contamination are shown in parenthesis and/or red arrows. [file Image_5.tif]
